# Blood-brain transfer of Pittsburgh compound B in humans

**DOI:** 10.3389/fnagi.2013.00070

**Published:** 2013-11-07

**Authors:** Albert Gjedde, Joel Aanerud, Hans Braendgaard, Anders B. Rodell

**Affiliations:** ^1^Department of Neuroscience and Pharmacology, University of CopenhagenCopenhagen, Denmark; ^2^Department of Nuclear Medicine and PET Centre, Aarhus University HospitalAarhus, Denmark; ^3^Center of Functionally Integrative Neuroscience, Faculty of Health, Aarhus UniversityAarhus, Denmark; ^4^Department of Neurology, McGill UniversityMontreal, QC, Canada; ^5^Department of Radiology and Radiological Science, Johns Hopkins UniversityBaltimore, MD, USA; ^6^Department of Neurology, Aarhus University HospitalAarhus, Denmark

**Keywords:** Alzheimers disease, blood-brain barrier, cerebral blood flow measurement, permeability-surface area product, pittsburgh compound B

## Abstract

In the labeled form, the Pittsburgh compound B (2-(4′-{N-methyl-[^11^C]}methyl-aminophenyl)-6-hydroxy-benzothiazole, [^11^C]PiB), is used as a biomarker for positron emission tomography (PET) of brain β-amyloid deposition in Alzheimer's disease (AD). The permeability of [^11^C]PiB in the blood-brain barrier is held to be high but the permeability-surface area product and extraction fractions in patients or healthy volunteers are not known. We used PET to determine the clearance associated with the unidrectional blood-brain transfer of [^11^C]PiB and the corresponding cerebral blood flow rates in frontal lobe, whole cerebral cortex, and cerebellum of patients with Alzheimer's disease and healthy volunteers. Regional cerebral blood flow rates differed significantly between the two groups. Thus, regional and whole-brain permeability-surface area products were identical, in agreement with the observation that numerically, but insignificantly, unidirectional blood-brain clearances are lower and extraction fractions higher in the patients. The evidence of unchanged permeability-surface area products in the patients implies that blood flow changes can be deduced from the unidirectional blood-brain clearances of [^11^C]PiB in the patients.

## Introduction

The amyloid cascade is the leading current explanation of the etiology of Alzheimer's disease (AD) (Hardy and Selkoe, 2002; Jack et al., 2010). The hypothesis holds that amyloid-β (Aβ) has a primary role in the biochemical, histological, and pathological changes that happen in the brain when AD evolves. In this process, the deposition of Aβ is considered an early event, which implies that biomarkers of Aβ may detect the presence of disease at the earliest stages of onset (McKhann et al., 2011).

Modification of the amyloid-binding dye thioflavin-T made non-invasive PET imaging possible with the labeled form of the Pittsburgh compound B, the tracer [^11^C]PiB. Although other tracers currently are under evaluation (Wong et al., 2013), [^11^C]PiB is the most extensively examined marker of Aβ in human studies with PET (Klunk et al., 2004; Price et al., 2005; Mintun et al., 2006; Lockhart et al., 2007; Ikonomovic et al., 2008). Compared to cognitively intact controls, AD patients exhibit greater [^11^C]PiB retention in areas known to have high Aβ deposits such as the frontal and parietal cortices, whereas brain areas relatively unaffected by Aβ pathology, such as the cerebellum, show equivalently modest [^11^C]PiB retention (Klunk et al., 2004; Price et al., 2005; Ikonomovic et al., 2008). However, the specific transport and binding properties of [^11^C]PiB remain uncertain when the tracer passes from the circulation into the tissue of the brain.

Klunk et al. (2004) compared the brain uptake of [^11^C]PiB with the uptake of fluorine-18-labeled fluorodeoxyglucose ([^18^F]FDG), a marker of glucose consumption in brain of patients with Alzheimer's disease. The authors found that [^11^C]PiB retention at late times correlated inversely with cerebral glucose metabolism traced by [^18^F]FDG. In the brain of patients with Aβ deposits, compared to patients without Aβ deposits, significant [^11^C]PiB retention correlated well with variable levels of amyloid deposition, as determined by later postmortem analysis (Klunk et al., 2004). However, at earlier circulation times, Rostomian et al. (2011) found comparable [^11^C]PiB and [^18^F]FDG uptakes in patients, consistent with substantial initial clearance of [^11^C]PiB from brains of patients, as also found in brains of healthy control subjects studied by Blomqvist et al. (2008). These results indicate that the phases of initial uptake and subsequent retention of [^11^C]PiB differ in human brain, with initial deposit of [^11^C]PiB limited by delivery and hence also by cerebral blood flow, coupled to metabolism, and later retention limited by binding to tissue components such as Aβ (Forsberg et al., 2012).

To determine the extent to which the initial uptake of [^11^C]PiB is flow-limited, and to convert the clearance to a measure of permeability, it is necessary to compute the permeability-surface area products of microvascular transport of [^11^C]PiB in different brain regions, as well as the fractions of extracted [^11^C]PiB. To obtain these values, we must know the values of the cerebral blood flow (CBF) that delivers [^11^C]PiB to brain tissue. Here, to test the hypothesis of initial flow-limited brain uptake of [^11^C]PiB in humans, we determined CBF values and [^11^C]PiB uptake in five patients with AD, and six age-matched healthy volunteers, by means of PET.

## Methods

### Subjects

Five patients with AD (3 women, 2 men) with an average age of 64 (*SD* = 7) years and moderately reduced Mini-mental State Examination (MMSE) scores of 22–25 volunteered to complete the tomography. The patients were recruited by the local Dementia Clinic and screened by an experienced neurologist for the presence of definite Dementia of Alzheimer's Type (DAT). Six healthy age-matched HC volunteers with MMSE scores in the range of 28–30 with a mean age of 70 (*SD* = 5) years served as controls, recruited by public advertisement and screened with clinical, neurological, and neuropsychological testing including MMSE adapted to Danish (Lolk et al., 2000) to exclude cognitive impairment. We obtained written informed consent from all subjects to the protocols approved by the Regional Science Ethics Committee in accordance with the Declarations of Helsinki. We previously reported PET results from the same five patients and from eight healthy volunteers, including the six healthy volunteers studied here (Rodell et al., 2012). We were unable to include two of the healthy volunteers because arterial samples of [^11^C]PiB concentration were unavailable.

### Positron emission tomography

#### Image acquisition

All subjects had positron emission recordings, one or two with [^15^O]water and one with [^11^C]PiB in the 3D mode of the ECAT High Resolution Research Tomograph (HRRT, CTI/Siemens, Knoxville, TN, USA) in a quiet room with the subjects resting in a supine position with eyes open. The images were reconstructed with three-dimensional ordinary Poisson ordered subset expectation maximization (3D-OP-OSEM) point spread function reconstruction (Varrone et al., 2009), using 10 iterations and 16 subsets with FWHM at approximately 1.5 mm. The reconstructed images were corrected for random and scatter events, detector efficiency variations, and dead time. Tissue attenuation scans were performed using a rotating 68Ge source. Dynamic emission recordings lasting 3 min (21 frames), or 90 min (30 frames) were initiated upon bolus intravenous injection of [^15^O]water (500 MBq) or [^11^C]PiB (500 MBq), respectively. Catheters (Artflon and Venflon, Becton Dickinson, Swindon, UK) were inserted in the right radial artery and left cubital vein. For the [^15^O]water sessions, arterial blood radioactivity was measured every half second for the duration of the PET scan by an automated blood sampling system (Allogg AB, Mariefred, Sweden), cross-calibrated with the tomograph, and then corrected for external delay and dispersion. For the [^11^C]PiB sessions, arterial blood radioactivity was measured in blood samples drawn manually for the duration of the tomography session. For anatomical orientation, high-resolution T1-weighted MR images were obtained at 1.5 or 3 T (GE Sigma Systems).

#### Image registration and segmentation

The summed emission recordings of [^15^O]water and [^11^C]PiB were automatically co-registered to the individual MRI scans using a 6 parameter affine transformation. Individual MR images initially were co-registered to a common stereotactic space (Montreal Neurological Institute) (Collins and Evans, 1997), using a 12-parameter affine rigid body transformation, and subsequently non-linearly transformed. After the calculation of the final PET-Talairach transformation matrix, dynamic emission recordings were re-sampled into common coordinates. Regional CBF and PiB permeability measures (see below) were obtained from parametric PET image maps using standard model based segmentation (Collins and Evans, 1997) into regions that included frontal lobe (FL), cerebral cortex (CTX), and cerebellum gray matter (CERB).

### Calculation of cerebral blood flow

We quantified the CBF as the water clearance *K*^H_2_O^_1_ in units of mL/100 g/min with the linearized two-compartment model (Blomqvist, 1984) modification of Ohta et al. (1996) and the Lawson–Hanson non-negative least squares solution to general least squares functions (Lawson and Hanson, 1974).

### Calculation of blood-brain transfer constants of PiB uptake

#### Unidirectional blood-brain clearance

We analyzed the uptake of [^11^C]PiB by means of multilinear graphical analysis (Gjedde, 1981, 1982; Patlak et al., 1983). From the time-radioactivity records in arterial blood samples and brain tissue, we constructed two derived variables, the dependent variable *V*(*T*) and the independent variable Θ(*T*), according to the principles of the Gjedde-Patlak Plot, as expanded by Gjedde and Wong (2001). The two variables are the time-dependent apparent volume of distribution,

(1) $$V(T)=\frac{m(T)}{c_{a}(T)}$$

and the apparent time of accumulation,

(2) $$\Theta (T)=\frac{\int_{o}^{T}c_{a}(t)\;dt}{c_{a}(T)}$$

which are related by the four parameters of the three-compartment model of irreversible accumulation, the net clearance *K*, the unidirectional clearance *K*_1_, the dynamic volume of the precursor compartment *V_g_*, and the dynamic volume of the vascular compartment *V_o_*,

(3) $$V(T)=K\;\Theta \text{​}(T)+V_{g}\text{​}\left(1-e^{-(K_{1}-K)\;\Theta \text{​}(T)/V_{g}}\right)+V_{o}$$

that for a simple reversible exchange between the vascular compartment and a single tissue compartment, for which *K* = 0 (*k*_3_ = 0), reduces to,

(4) $$V(T)=V_{g}\text{​}(1-e^{-K_{1}\Theta \text{​}(T)/V_{g}})+V_{o}$$

used here to obtain the estimates of the unidirectional clearance *K*_1_, because the regression of the complete equation (3) yielded estimates of *K* that were not significantly different from zero. At early times (Θ(*T*) → 0) *V*(*T*) approaches the linear relationship (Gjedde, 1981),

(5) $$V(T)\to K_{1}\;\Theta \text{​}(T)+V_{o}$$

#### Permeability and extraction fraction

The values of cerebral blood flow (*F*), unidirectional clearance (*K*_1_), and permeability-surface area product (*PS*) are related by the formula (Crone, 1963, 1965; Gjedde and Christensen, 1984),

(6) $$K_{1}=F\left(1-e^{-PS/F}\right)$$

which yields the extraction fraction as the ratio between the unidirectional clearance and the blood flow,

(7) $$E=K_{1}/F=1-e^{-PS/F}$$

from which derives the permeability-surface area product,

(8) PS=−Fln(1−E)

## Results

### Arterial plasma concentrations

**Figure 1:**
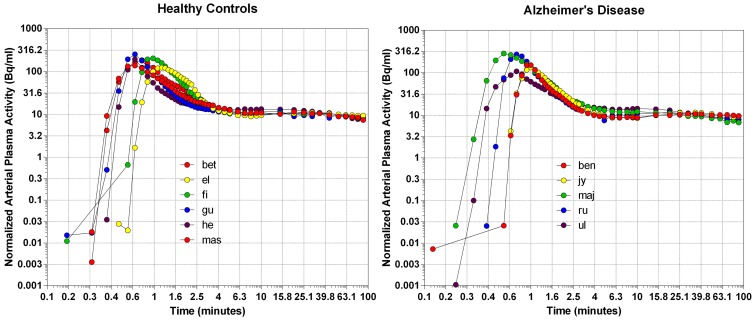
**The distributions of arterial plasma concentrations in healthy volunteers (left panel) and patients with Alzheimer's disease (right panel), normalized to average Area Under Curve (AUC)**. Abscissa: Log10 rescaling of Time (minutes). Ordinate: Log10 rescaling of average normalized arterial plasma radioactivity.

We normalized the arterial plasma concentrations of [^11^C]PiB to the area under the curve, as shown in Figure 1. The concentrations revealed a triphasic time course, with an initial rapid rise to a common peak within a minute of administration, an immediate decline to a plateau at 4 min, maintained at about 10% of the peak activity until the end of sampling at 90 min. The plateau represents metabolites of [^11^C]PiB, appearing in significant amounts after about 4 min of circulation of the tracer, as shown in Figure 1. When the arterial samples are corrected for the metabolite fractions determined by Lopresti et al. (2005), the unchanged tracer [^11^C]PiB had declined to less than 3% of the peak activity by 10 min after the administration, and to 1% by 15 min, indicating that the tracer practically had disappeared from the circulation at that time.

### Brain time-activity curves

**Figure 2:**
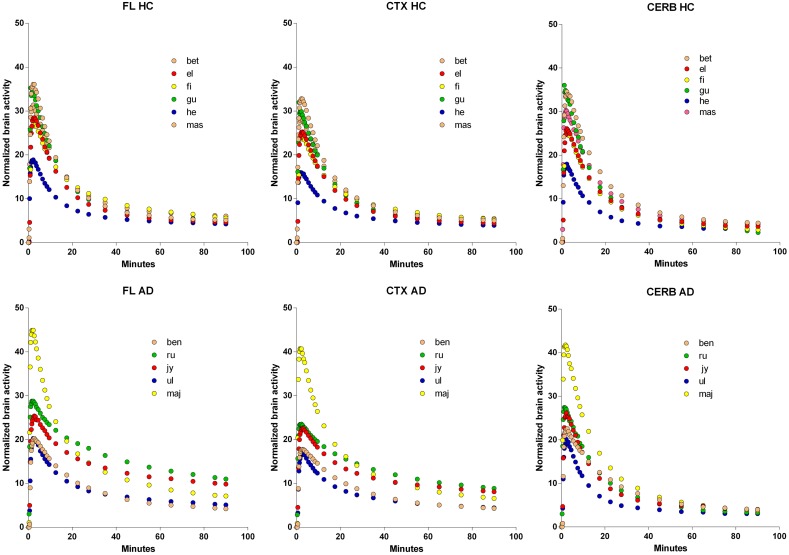
**Normalized individual Time-Activity Curves of three brain regions, Frontal Lobe (FL), Cerebral Cortex (CTX), and Cerebellum (CERB), in 6 healthy volunteers (upper row), and 5 patients with Alzheimer's Disease (lower row)**. Abscissae: Time (minutes). Ordinates: Normalized brain radioactivity (kBq/ml).

**Figure 3:**
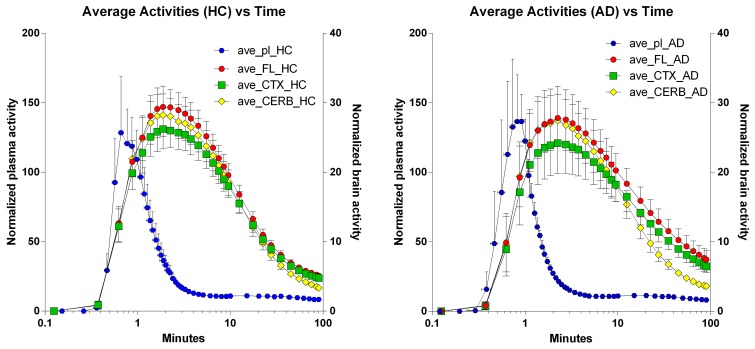
**Average regional brain Time-Activity Curves superimposed on average arterial concentration curves. Left panel:** Healthy volunteers. **Right panel:** Patients with Alzheimer's disease. Abscissae: Time (minutes, log10 scale). Left ordinates: Average normalized plasma activity (kBq/ml). Right ordinates: Average normalized brain activity (kBq/cm^3^).

The time-activity curves of the three brain regions selected for the two groups of subjects indicate rapid uptake of [^11^C]PiB, which peaks at approximately 2 min, and subsequent more or less rapid washout from about 4 min onwards, as shown for the individual subjects in Figure 2, and for the averages in Figure 3, superimposed on the average arterial concentration curves.

### Multitime graphical analysis

**Figure 4:**
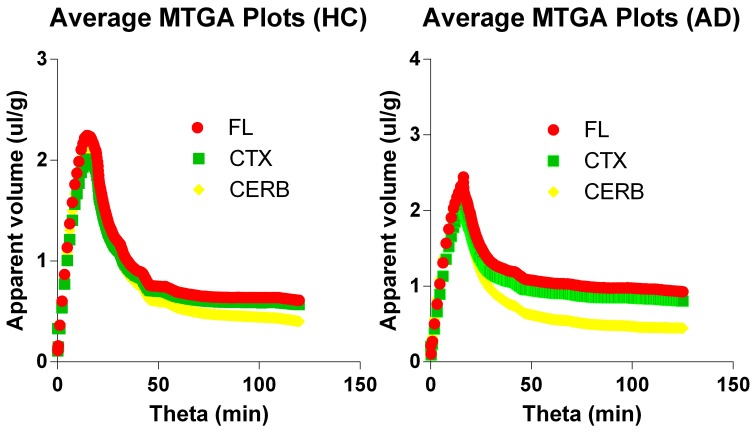
**Multitime graphical analysis (MTGA) plots of blood-brain transfer of [^11^C]PiB according to Equations (1) and (2)**. MTGA plots reveal effect of generation of metabolites that do not enter brain tissue and disappearance of [^11^C]PiB from circulation, with rapid reduction of apparent volume of distribution [^11^C]PiB in brain after 4 minutes of circulation, corresponding to 10 min in units of normalized time. **Left panel:** Healthy volunteers. **Right panel:** Patients with Alzheimer's disease. Abscissae: Normalized time (minutes). Ordinates: Apparent volume of distribution (ml/cm^3^).

**Figure 5:**
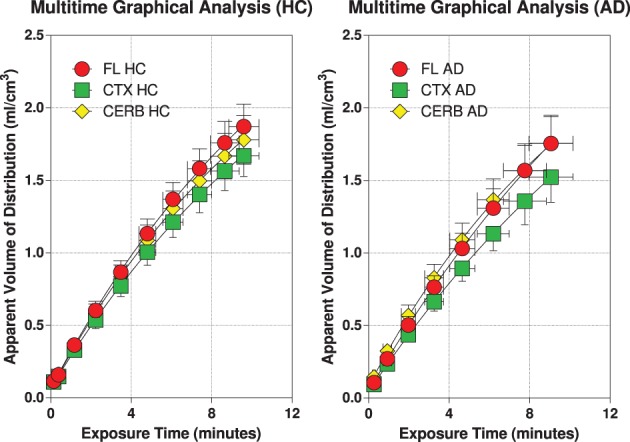
**Individual MTGA or Gjedde-Patlak plots with regression curves according to Equations (1) and (2) for 6 healthy volunteers (left panel), and 5 patients with Alzheimer's disease (right panel)**. Abscissae: Exposure or ‘Theta’ (defined in Equation 2) (“normalized”) time (minutes). Ordinates: Apparent volume of distribution (ml/cm^3^).

**Figure 6:**
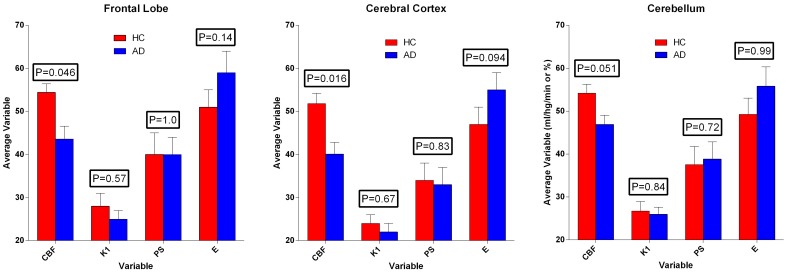
**Average estimates (± SEM) of cerebral blood flow and unidirectional clearance, and the calculated values of extraction fraction and permeability-surface area product of [^11^C]PiB, according to Equations (7) and (8)**. Values of CBF exceeded values of PS-products, which in turn varied among regions, but did not differ significantly between the two groups. Ordinates: Units of flow (ml g^−1^ min^−1^) and extraction percentage (%).

The multitime graphical analysis (MTGA) or Gjedde-Patlak plots according to equations (1) and (2) show the clear effect of the generation of metabolites of [^11^C]PiB that do not enter brain tissue, with rapid reduction of the apparant volume of distribution of [^11^C]PiB in brain tissue as a result, as shown in Figure 4. For this reason, we limited the period of analysis of blood-brain transfer of [^11^C]PiB to the period 0–4 min, when the generation of metabolites was negligible. The MTGA plots for the time periods 0–4 min are shown for patients and control subjects in Figure 5, in which the regression curves are shown. When analyzed according to equation (4), the regression yielded consistent estimates of the four parameters of equation (3), with values of *K* close to zero, indicating single tissue compartment kinetics (*k*_3_ ≅ 0) according to equation (4). With the value of *K* fixed at zero, the analysis yielded the estimates of *K*_1_, *V_g_*, and *V_o_*, shown in Figure 6, together with the estimated values of cerebral blood flow in the same regions. While the regional CBF values differed significantly between the two groups, the unidirectional clearances numerically varied in the same direction as the blood flow values but the differences were not significant.

### Permeability-surface area products

We used the estimates of unidirectional clearance and cerebral blood flow to calculate the extraction fractions and permeability-surface area products of [^11^C]PiB according to equations (7) and (8), as listed in Table 1 and shown in Figure 6. The values of the PS-products were lower than the CBF values and varied among regions, but the regional values did not differ significantly between the two groups.

**Table 1:** **Average estimates (± SEM) of unidirectional blood-brain clearance (*K*_1_), extraction percentage (*E*), and permeability-surface area product (*PS*) in regions of brain of healthy control subjects (HC) and patients with Alzheimer's disease (AD)**.

**Variable**	**Unit**	**Frontal lobe**			**Cortex**			**Cerebellum**		
		**HC (*n* = 6)**	**AD (*n* = 5)**	***P***	**HC (*n* = 6)**	**AD (*n* = 5)**	***P***	**HC (*n* = 6)**	**AD (*n* = 5)**	***P***
*F*	ml hg^−1^ min^−1^	54 ± 2	43 ± 3	0.046	52 ± 2	40 ± 3	0.016	54 ± 2	47 ± 2	0.051
*K*_1_	ml hg^−1^ min^−1^	28 ± 3	25 ± 2	0.57	24 ± 2	22 ± 2	0.67	27 ± 2	26 ± 2	0.84
*E*	%	51 ± 4	59 ± 5	0.14	47 ± 4	55 ± 4	0.094	49 ± 4	56 ± 4	0.99
*PS*	ml hg^−1^ min^−1^	40 ± 5	40 ± 4	1.0	34 ± 4	33 ± 4	0.83	38 ± 4	39 ± 4	0.72

P-values indicate probability of insignificant difference, with correction for multiple comparisons.

## Discussion

In previous publications, we determined the variability of cerebral blood flow rates in patients with Alzheimer's disease (Rodell et al., 2012), and we determined the accumulation of tracer [^11^C]PiB in regions of the brains of the same patients in relation to cerebral blood flow, compared to healthy control subjects.

The issue resolved in the present report is the question of whether [^11^C]PiB uptake into the brains of these patients and healthy volunteers during the early phase of blood-brain transfer is flow-limited such that the initial uptake of [^11^C]PiB can be used reliably as a marker of blood flow. The evidence from the study supports the claim that [^11^C]PiB uptake during the first 4 min of circulation is not completely flow-limited, consistent with substantial but not unlimited permeability in the cerebral vasculature of patients with Alzheimer's disease and healthy control subjects. The PS-products were less than the values of cerebral blood flow, establishing unidirectional extraction fraction values at 0.63 or less. A tracer with this property yields flow values that are lower than the true values, and the flow fluctuations are attenuated. Thus, the estimated PS-products may not be sufficiently high to secure unidirectional clearance rates that always differ significantly when blood flow rates decline.

The search for tracers with initial flow-limited uptake sufficient to accurately estimate blood flow rates has been ongoing for a long time. The issue always concerned the duration of the flow-limited accumulation, and the determination of the moment when the accumulation effectively turns from flow-limitation to significant limitation by diffusion, binding, or metabolism. This transition happens for all tracers, given enough time. The use of the marker to trace blood flow therefore is limited to the period before the transition.

**Figure 7:**
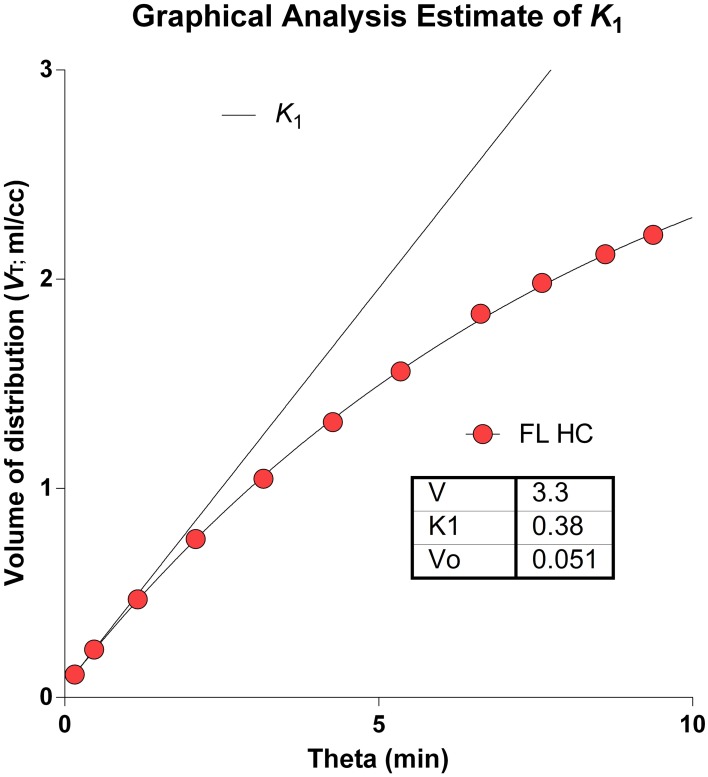
**Idealized clearance and actual uptake of [^11^C]PiB into frontal lobe of healthy volunteer**. Determination of brain microvascular Permeability-Surface Area products (PS) rests on measure of initial blood-brain transfer, or clearance, symbolized by term *K*_1_. Abscissa: Normalized time ‘Theta’ (minutes) as defined in Equation 2. Ordinate: Apparent volume of distribution (ml/cm^3^). Note that [^11^C]PiB uptake into brain tissue is partially flow-limited for only a few moments after administration.

The determination of the brain's microvascular PS-products rests on the measure of the marker's initial clearance, here symbolized by the term *K*_1_, as shown in Figure 7, displaying both the initial idealized clearance, as derived from the actual uptake of [^11^C]PiB into the frontal lobe of a healthy volunteer. Judging from this graph, [^11^C]PiB uptake into brain tissue is effectively unidirectional for only a few minutes after administration the tracer. The accurate determination of this initial clearance is aided by regression to the measured monoexponential approach to a steady-state volume of distribution.

In previous attempts to obtain blood-brain clearance measures of [^11^C]PiB, the authors generally concluded that clearances and hence uptake rates are “high” but values of permeability-surface areas were not reported (Blomqvist et al., 2008; Rostomian et al., 2011), although Blomqvist et al. did obtain unidirectional clearance estimates. Blomqvist et al. (2008) assumed continuous steady-state accumulation of [^11^C]PiB in brain tissue, for which there is little evidence. The issue reflects the uncertainty of the number of compartments of [^11^C]PiB distribution in brain tissue that separately and significantly can be identified kinetically. The present analysis gave no evidence of continuous steady-state accumulation of [^11^C]PiB or its labeled metabolites in brain tissue and instead reflected the kinetics of a single tissue compartment.

There is no strongly correlated decline of brain energy metabolism and cerebral blood flow rates in healthy aging (Aanerud et al., 2012). Neurodegeneration, on the other hand, such as Alzheimer's disease, generally presents with declines of both variables in specific parts of the brain, most likely driven by the reduced energy turnover in these same parts of the brain of demented individuals (Johannsen et al., 2000; Rodell et al., 2012). The unidirectional blood-brain clearance term *K*_1_ is an expression of the unidirectional influx of [^11^C]PiB and as such is related to the blood flow rate by means of equation (6). Therefore, we expected to find a correlation between blood flow estimates and [^11^C]PiB accumulation in the first tomography frames of [^11^C]PiB uptake. This expectation was supported by the inverse correlation between blood flow and [^11^C]PiB accumulation in the last tomography frames, as evidence of the presence of Aβ plaques in the tissue. Indeed, blood flow and [^11^C]PiB accumulation at that time were highly correlated (*R*^2^ = 0.999; *P* = 0.021).

To convert estimates of unidirectional clearance to blood flow values, the extraction fraction must be known, according to the equation, *F* = *K*_1_/*E*. In the present study, the unidirectional extraction fraction averaged 0.53 for all subjects and regions, but differed significantly between the patients and the healthy control subjects. In a companion study, we used the unidirectional clearance and estimates of PS products to make accurate predictions of blood flow that correlated well with actual CBF measures. With an average extraction fraction of 0.53, the values of blood flow calculated from the unidirectional clearance respectively over- and underestimated the CBF values determined with labeled water in patients and healthy control subjects and did not differ significantly on average.

No non-gaseous cerebral blood flow indicator is fully extracted. Depending on the distribution volume, a quantity of tracer always remains in the vascular bed, implying that there is no perfect single compartment modeling that fully describes the kinetics (Ohta et al., 1992, 1996). The conversion of non-gaseous tracer uptake therefore depends on the validity of the extraction fraction used to calculate the blood flow from the unidirectional blood-brain clearance, determined kinetically or graphically. The extraction fractions for typical non-gaseous blood flow indicators vary from more than 90% for ethanol, iodoantipyrine, and butanol, to less than 90% for antipyrine and water (Gjedde et al., 1975, 1980; Sakurada et al., 1978). The lower the extraction fraction, the more the calculation itself depends on the unknown blood flow.

**Figure 8:**
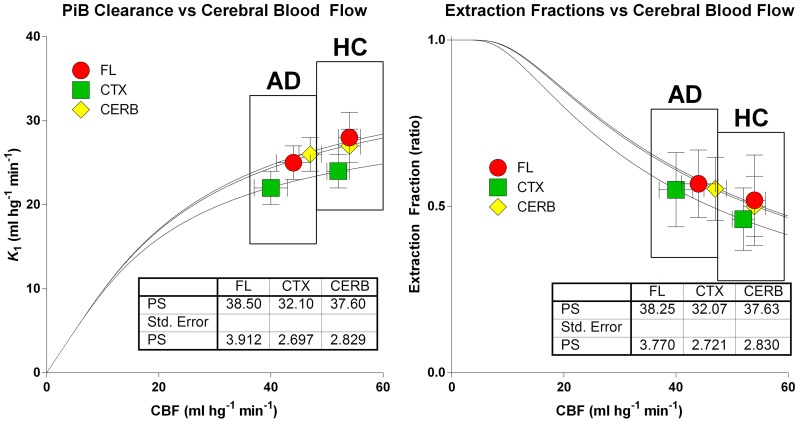
**Relations among clearance (left panel) and extraction fraction (right panel) estimates and measured cerebral blood flow values in patients and healthy control subjects according to equations (6) and (7)**. Abscissae: Cerebral Blood Flow estimates (ml hg^−1^ min^−1^). Ordinate **(left panel)**: Unidirectional [^11^C]PiB Clearance (ml hg^−1^ min^−1^). Ordinate **(right panel)**: Extraction fraction (ratio). Similar regional PS-products in the two groups explain numerically attenuated and insignificant differences of unidirectional clearances and extraction fractions.

**Figure 9:**
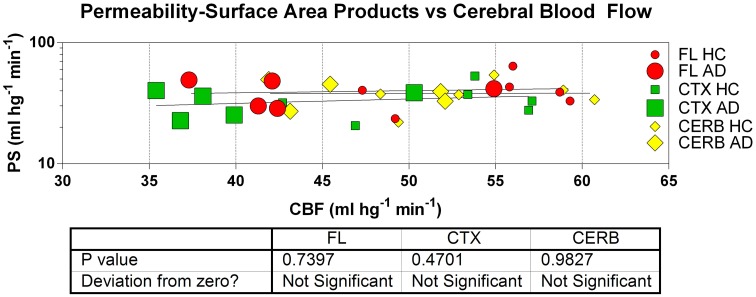
**Estimates of permeability-surface area (PS) products reveal no relation to measures of cerebral blood flow (CBF) in frontal lobe (FL), cerebral cortex (CTX), and cerebellum (CERB) of patients and healthy control subjects**. Abscissa: CBF (ml hg^−1^ min^−1^). Ordinate: PS products (ml hg^−1^ min^−1^). Absent correlation is consistent with incomplete flow-limitation of blood-brain transfer of [^11^C]PiB.

The relations among the clearance and extraction estimates and the measured flow values are illustrated in Figure 8. The similar regional PS-products in the two groups explain the numerically attenuated and hence insignificant differences between the extraction fractions. Thus, the comparatively low PS-products are consistent with extraction fractions that do not change very significantly with changing blood flow rates, as shown in Figure 9.

**Figure 10:**
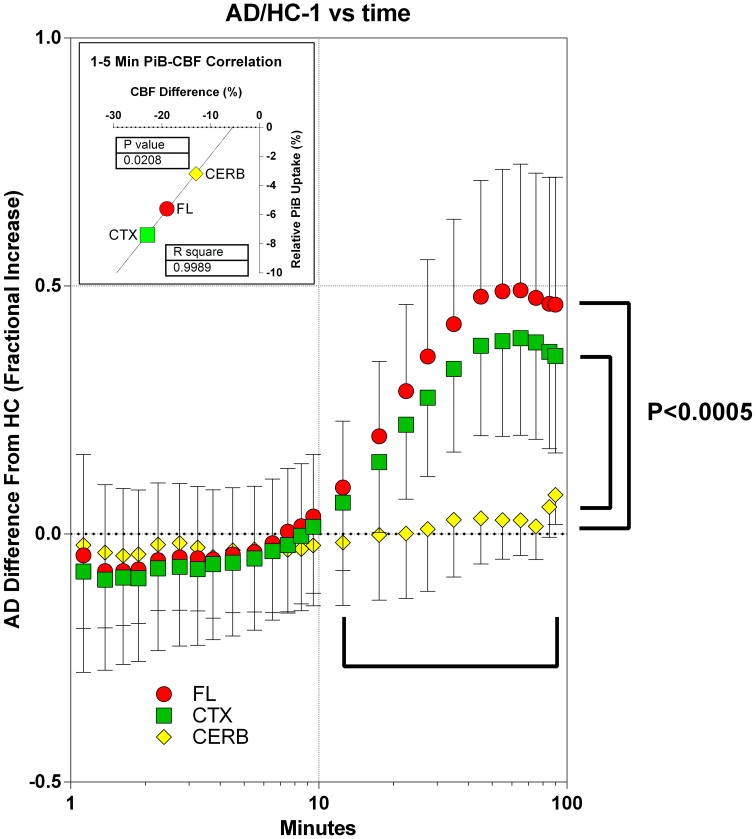
**Accumulation of [^11^C]PiB in brain tissue in periods of 1–10 min and after 10 min, in patients in relation to healthy volunteers**. Abscissa: Time (minutes, log10 scale). Ordinate: Fractional difference of accumulation between patients and control subjects (ratio - 1). From 1 to 5 min of circulation, brain uptake of [^11^C]PiB relative to healthy controls correlated significantly with the differences of cerebral blood flow (*R*^2^ = 0.999; *P* = 0.021, data not shown). After 10 min of circulation among patients and healthy controls, relative uptake differed significantly (*P* < 0.0005) for frontal lobe and cerebral cortex, but not for gray matter of cerebellum. In cortical regions, uptake of [^11^C]PiB in patients exceeded that of healthy controls by 40–50%, indicating binding-limited [^11^C]PiB accumulation in this period. For the cerebellum, the accumulation of [^11^C]PiB did not differ between the two groups.

The information that can be gleaned from the [^11^C]PiB uptake clearly depends on when the uptake is determined. After 10 min of circulation, among the two groups, the retention of [^11^C]PiB and its possible metabolites differed significantly for the frontal lobe and cerebral cortex, but not for the gray matter of the cerebellum. In the cortical regions, the uptake of [^11^C]PiB in the patients with Alzheimer's disease exceeded that of the healthy controls by 40–50%, indicating [^11^C]PiB retention at this time. For the cerebellum, the accumulation of [^11^C]PiB did not differ between the two groups, as shown in Figure 10. From 1 to 4 min of circulation, the brain uptake of [^11^C]PiB relative to healthy controls correlated significantly with the differences of cerebral blood flow (*R*^2^ = 0.999; *P* = 0.021), indicating the partially flow-limited uptake of [^11^C]PiB during this period shown in Figure 10.

To claim that a tracer with the properties of [^11^C]PiB with an average extraction fraction of 0.53 ± 0.10 (SD), is useful as a flow indicator at the very least requires a careful assessment of the variability of the results and the potential errors that may arise. In the present study, the correlation coefficient of measured and calculated flow values was *R*^2^ = 0.20 with a significance of *P* < 0.01 and a slope of 58% of the line of identity for a total of 33 comparisons (3 brain regions from 11 subjects each).

### Conflict of interest statement

The authors declare that the research was conducted in the absence of any commercial or financial relationships that could be construed as a potential conflict of interest.
